# Impacts of inflammatory cytokines on depression: a cohort study

**DOI:** 10.1186/s12888-024-05639-w

**Published:** 2024-03-08

**Authors:** Fei Liu, Yang Yang, Xiao-Wei Fan, Ning Zhang, Shuo Wang, Yi-Jun Shi, Wei-Jiang Hu, Chun-Xue Wang

**Affiliations:** 1 Department of neurology, Hangzhou First People’s Hospital, Hangzhou, Zhejiang China; 2 Department of Neuropsychiatry and Behavioral Neurology and Clinical Psychology, Beijing Tiantan Hospital, Capital Medical University, 119 South 4th Ring West Road, Beijing, 100070 China; 3 Department of neurology, Beijing Tiantan Hospital, Capital Medical University, Beijing, China; 4 Department of Clinical Diagnosis Laboratory, Beijing Tiantan Hospital, Capital Medical University, Beijing, China

**Keywords:** Plasma cytokine, Depression, Suicidal ideation and behavior, Social support

## Abstract

**Background:**

Inflammatory factors are associated with depression. We seek to investigate the correlation between inflammatory cytokines and prognosis of depression or suicidal ideation and behavior at 3 months in depression patients.

**Methods:**

Eighty-two depressed outpatients were recruited and treated as usual. Plasma cytokines were measured at baseline. Patients were followed up with Patient Health Questionnaire-9 and suicidal ideation and behavior according to the item 3 of Hamilton depression scale for 3 months.

**Results:**

Compared to the depression patients with low level of interleukin-1β, the high one had severe depressive symptoms at month 2 and 3 (B 0.92, *P* < 0.01; B 0.86, *P* = 0.02; respectively). The incidence of suicidal ideation or behavior was 18.3% at 3 months. Depression patients with high levels of tumor necrosis factor-α showed high risk of suicidal ideation and behavior than the low one (OR 2.16, 95% CI 1.00-4.65, *P* = 0.04).

**Conclusions:**

High levels of interleukin-1β and tumor necrosis factor-α were predictive of middle-term severe depressive symptoms and suicidal ideation and behavior respectively.

**Supplementary Information:**

The online version contains supplementary material available at 10.1186/s12888-024-05639-w.

## Background

Depression affects 4.4% of the population and is the leading cause of disability globally [1]. Approximately one-third of depression patients fail to remit with adequate dose and duration antidepressant treatment [2]. The global suicide rate is really high and results in the loss of nearly one million lives each year [3]. About 37.7% and 15.1% depression patients have suicidal ideation and behavior [4]. The mechanisms of good prognosis for depression and depressive patients accompanied with suicide are not entirely clear, which indicates the urgent need to develop novel strategies.

Inflammatory system plays a complicated role in depression. Plasma cytokines, a family of polypeptides, include interleukin (IL), interferon (IFN), tumor necrosis factor (TNF), tumor transforming growth factor (TGF), etc. Cytokines are generally divided into pro-inflammatory (IL-1β, IL-2, IL-6, IL-12, IL-15, TNF-α, IFN-γ, etc.) and anti-inflammatory cytokines (IL-4, IL-5, IL-10, IL-13, etc.) [5]. Some pro-inflammatory cytokines are upregulated in depression people [6], and also positively correlated with the severity of depressive symptoms [7].

Cytokines imbalance causing dysregulation of hypothalamic-pituitary-adrenal (HPA) axis and neurogenesis is considered to be a key factor in the development and treatment of depression [8]. Plasma cytokines can penetrate the blood brain barrier (BBB) to affect brain function directly or activate microglia in the central nervous system (CNS), and also communicate with the neuroendocrine system by transmitting signals to the hypothalamus and stimulates the release of corticotropin releasing hormone (CRH), adrenocorticotropic hormone (ACTH) and glucocorticoids ultimately [9, 10]. Glucocorticoids lead to neuronal atrophy in the depression related prefrontal cortex and hippocampus [11]. Cytokines are involved in glutamatergic and monoamine neurotransmission in the CNS. They reduce the level of glutamate transporters and increases the level of glutamate [12]. Glutamatergic synapses are associated with depression related cerebral regions, including prefrontal cortex and hippocampus [11]. Pro-inflammatory cytokines regulate 5-hydroxytryptamine (5-HT) turnover in brain and decrease levels of 5-HT in the synaptic cleft, which may influence neuroplasticity and finally result in depression [13, 14]. They also inhibit neurogenesis by activating nuclear factor κB [15].

The behavioral effects of serotonergic antidepressants or probiotics may depend on regulating cytokines formation partly. Anti-inflammatory agents antagonize depressive behavioral responses to selective serotonin reuptake inhibitors (SSRIs), TNF-α and INF-γ may be involved in this progress [16]. After consuming Lacticaseibacillus paracasei strain Shirota for 9 weeks, the depressive symptoms are significantly improved accompanied by a decrease in levels of IL-1β, IL-6 and TNF-α [17]. In some studies, cytokine inhibitor therapies, such as anti-IL-6 biologics, TNF-α inhibitors, IL-12 and IL-23 antagonists were efficacious in depression [18, 19]. IL-10, anti-inflammatory cytokine, reverses depression-like behavior [20]. After treatment with interferon and ribavirin, there was positive correlation between depression severity and cytokines including IL-8, IL-10, IL-16, TNF-α, TGF-β and IFN-β in patients with chronic hepatitis C. Both pro- and anti-inflammatory responses were activated [21]. The improvement of depressive symptoms after treatment of antidepressants is related to the reduction of cytokines including IL-1β and IL-6 [22]. Targeting inflammation may be a potential strategy to treat depression.

Suicide has not been completely understood from an etiological perspective. HPA stress-response system, monoaminergic neurotransmitter systems and brain-derived neurotrophic factor play an important role in suicide cases [23]. Depression patients with suicidal attempts have high levels of blood pro-inflammatory cytokines such as TGF-β, CRP, and decreased levels of anti-inflammatory cytokines such as IL-4 [24, 25]. They also exhibit increased in IL-1β, IL-6, TNF-α levels and decreased IL-10, IL-1 receptor antagonists levels in the prefrontal cortex [18].

Numerous proteins in peripheral blood can be used as markers of systemic inflammation. In this report, we aimed to explore the association between cytokines and later severity of depression or suicidal ideation and behavior, which may be potential treatable targets of depression.

## Methods

### Study settings and participants

This is a single-site cohort study designed to examine the relationship between plasma cytokines and depression disorders. Participants included outpatients treated at the Beijing Tiantan Hospital, Capital Medical University from January 2020 to September 2020.

All participants met the inclusion criteria: (1) age over 18; (2) diagnosis of depression by a psychiatrist using Diagnostic and Statistical Manual of Mental Disorders-V criteria. Patients in the following criteria were excluded: (1) accompanied by other mental diseases such as schizophrenia and bipolar disorder (except for anxiety disorder); (2) have a history of infection, antibiotics or anti-inflammatory medications within a month; (3) have autoimmune disease or long time use of immunosuppressive drugs (such as steroids, cyclophosphamide, monoclonal antibodies, etc.).

### Data collection

Social-demographic information, medical history, depression disorder, scale measurements (social support rate scale, life-change-unite-score and Patient Health Questionnaire-9 (PHQ-9)) assessed by psychiatrists and blood samples were collected at baseline face-to-face. PHQ-9 was assessed online at month 1, 2 and 3. Suicidal ideation and behavior was collected at month 3.

Social demographic variables included age, gender, body mass index (BMI), marital status (married, others), education (below high school, high school and above), employment status (employed, others), smoking and alcohol use (yes, no), income (< 50,000, 50,000-100,000, > 100,000 RMB per year, unknown), family history of mental illness (yes, no), physical activity (none, minor, moderate, severe).

Physical activity = intensity × time × frequency, includes four grades: (1) none (≤ 4 points); (2) minor (5–19 points); (3) moderate (20–42 points); (4) severe (≥ 43 points).

Intensity includes five grades: (1) mild exercise (walking etc.), 1 point; (2) low intensity exercise (table tennis, jogging, Taiji etc.), 2 points; (3) moderate intensity exercise (running, bicycle riding etc.), 3 points; (4) high intensity exercise with short time (badminton, volleyball, basketball, tennis etc.), 4 points; (5) high intensity exercise with long time (running race, swimming etc.), 5 points.

Time includes five grades: (1) ≤ 10 min, 1point; (2) 11–20 min, 2 points; (3) 21–30 min, 3 points; (4) 31–59 min, 4 points; (5) ≥ 60 min, 5 points.

Frequency includes five grades: (1) ≤ 1 time/month, 1point; (2) 2–3 times/month, 2 points; (3) 1–2 times/week, 3 points; (4) 3–5 times/week, 4 points; (5) about 1 time/day, 5 points.

Medical history variables included hypertension, hyperlipidemia, diabetes, heart disease, brain stroke, tumor, insomnia and sleep apnea hypopnea syndrome.

Depression situation included antidepressant drugs treatment, psychotherapy and the duration of depression.

Social support was measured by the social support rate scale designed for Chinese people by Xiao in 1986, with 10 items and a total score of 40 points. This scale mainly evaluates the support from family, friends, colleagues and society in terms of economy, problem-solving, and emotions. The higher score means greater support.

Life stress was measured by the life-change-unite-score, which is used to evaluated the past year situation and has 43 items. The higher LCU-score was associated with higher risk of multiple sclerosis and ulcerative colitis [26].

PHQ-9 was used to evaluate depressive severity, with a total score of 27 points. The PHQ-9 scores were determined at the time of the baseline assessment and months one, two and three [27].

Suicidal ideation and behavior was defined as the item 3 of Hamilton depression scale ≥ 1, which has 5 levels, from 0 to 4. No suicidal ideation and behavior, 0 point; Not worth living, 1 point; wishing he were dead, 2 points; suicidal ideation and half-hearted attempts, 3 points; severe attempts, 4 points [28].

Fourteen cytokines, i.e. IL-1β, IL-2, IL-4, IL-5, IL-6, IL-8, IL-10, IL-12P70, IL-17 A, IL-17 F, IL-22, TNF-α, TNF-β, IFN-γ, were assessed in fasting venous blood samples obtained in the morning after an overnight fast, and centrifuged at 3000 rpm/min for 20 min immediately. The plasma was kept frozen at -80 °C, and analysed by the flow cytometry.

### Statistical analysis

Descriptive data were presented as means ± standard deviation or medians (interquartile range, IQR) for continuous variables and counts (frequency percentages) for categorical variables. The One-way ANOVA or Kruskal-Walls test and Chi-square test or Fisher’s exact probability test were used for continuous and categorical variables separately.

Linear regression analysis was performed to evaluate the independent association between cytokines at baseline and depressive severity at month 1, 2 and 3. The results were presented with B and *P* value. The variance inflation factor (VIF) value of any variable exceeding 10 indicated the presence of multicollinearity. None was adjusted in Model 1. And model 2 adjusted the factors with *P* values < 0.2 in monofactor analysis.

Multivariate logistic regression analysis was performed to evaluate the correlation between the potential factors and suicidal ideation or behavior at month 3. Variables adjusted in the model included the factors with *P* values < 0.2 in monofactor analysis. The multivariate analysis results were presented as odds ratios (OR) [95% confidence interval] and *P* value.

All *p*-values were two-sided, with *p*-values < 0.05 considered statistically significant. All data analyses were conducted using SPSS statistical software version 25.

## Results

Of the eighty-two depression patients recruited, twenty-six were male, and fifty-six were female. They were all Chinese and between 18 and 67 years old. The average age was 37. Seventy-two, seventy-one and seventy-one patients were followed and assessed at month 1, month 2, and month 3, respectively. Thirteen (18.3%) patients had suicidal ideation or behavior at a 3-month follow-up. One of the patients had suicidal behavior. About half of the patients took SSRIs, and few patients took serotonin and norepinephrine reuptake inhibitors (SNRIs), norepinephrine and dopamine reuptake inhibitors (NDRIs), serotonin antagonists and reuptake inhibitors (SARIs), noradrenergic and selective serotonergic antidepressants (NaSSAs) and St John’s wort during follow-up. And the average PHQ-9 score were 12.4, 9.3, 7.9 and 7.4 at baseline, minth1, 2 and 3 separately.

### Impact of cytokines at baseline on depression at 3 months

At 1 month after enrollment, the severity of depression was related to IL-1β (B 0.75, *P* = 0.04), IL-2 (B 1.41, *P* = 0.01) and IL-4 (B 0.91, *P* = 0.01). While none of the cytokines was associated with depressive severity after adjustment in Model 2. At 2 and 3 months, only IL-1β was related to depressive severity in Model 1 (B 1.07, *P* < 0.01; B 1.05, *P* < 0.01; respectively). After adjusting for age, BMI, education, antidepressant drugs treatment, social support, life stress and PHQ-9 at baseline in model 2, IL-1β was still predictive of depressive severity (B 0.92, *P* < 0.01; B 0.86, *P* = 0.02; respectively) (Table 1). The VIF values for all included variables were less than 10 (Supplemental Tables 1, 2). PHQ-9 at baseline was predictive of depressive severity at month 2 (B 0.34, *P* < 0.01) and month 3 (B 0.32, *P* < 0.01) (Supplemental Tables 1, 2).

**Table 1:** Impact of cytokines on depression within 3 months

	PHQ-9											
	Month 1				Month 2				Month 3			
	Model 1		Model 2		Model 1		Model 2		Model 1		Model 2	
	B	P	B	P	B	P	B	P	B	P	B	P
IL-1β	0.75	0.04*	0.43	0.14	1.07	< 0.01*	0.92	< 0.01*	1.05	< 0.01*	0.86	0.02*
IL-2	1.41	0.01*	0.76	0.11	0.28	0.63	-0.22	0.70	0.11	0.86	-0.56	0.37
IL-4	0.91	0.01*	0.43	0.15	0.47	0.22	0.12	0.74	0.45	0.26	0.04	0.91
IL-5	0.94	0.17	0.52	0.31	-0.23	0.73	-0.60	0.33	-0.40	0.58	-0.64	0.34
IL-6	0.02	0.82	0.05	0.52	0.01	0.87	0.06	0.54	-0.02	0.81	-0.01	0.92
IL-8	-0.01	0.50	0.00	0.93	0.00	0.76	0.00	0.78	0.00	0.70	0.00	0.92
IL-10	0.41	0.16	0.31	0.16	-0.04	0.89	-0.12	0.64	-0.12	0.69	-0.17	0.56
IL-12P70	1.88	0.21	1.30	0.28	0.02	0.98	-0.55	0.69	-0.11	0.94	-1.04	0.50
IL-17 A	0.53	0.05	0.24	0.25	0.13	0.62	-0.07	0.77	0.11	0.70	-0.13	0.63
IL-17 F	0.16	0.83	0.00	0.99	0.46	0.54	0.38	0.57	-0.12	0.87	-0.27	0.71
IL-22	0.08	0.50	0.06	0.47	0.08	0.48	0.06	0.55	0.02	0.85	-0.01	0.92
TNF-α	0.30	0.39	0.27	0.30	0.43	0.47	0.71	0.22	0.49	0.43	0.45	0.47
TNF-β	0.68	0.26	0.38	0.42	0.32	0.59	0.03	0.94	0.38	0.55	0.15	0.79
IFN-γ	0.98	0.18	0.34	0.54	0.71	0.33	0.27	0.69	0.51	0.50	-0.14	0.84

Model 1: adjust for nothing

Model 2: adjust for age, BMI, education, antidepressant drugs, social support, life stress and PHQ-9 at baseline

PHQ-9, Patient Health Questionnaire-9; BMI, body mass index

*P < 0.05

### Impact of factors on the suicidal ideation and behavior at 3 months

Of the seventy-one patients, thirteen (18.3%) had suicidal ideation or behavior within 3-month follow-up. One of the patients had suicidal behavior. After adjusting age, gender, BMI, smoking use, antidepressant drugs, social support and PHQ-9 at baseline, TNF-α was at risk for suicidal ideation and behavior (OR 2.16, 95% CI 1.00-4.65, *P* = 0.04), while IL-1β was not (OR 1.26, 95% CI 0.90–1.78, *P* = 0.17). In addition, better social support predicted a low risk of suicidal ideation and behavior (OR 0.86, 95% CI 0.74–0.99, *P* = 0.04) (Table 2).

**Table 2:** Logistic regression analysis for the baseline TNF-α or IL-1β and suicide at month 3

	3-month suicide					
	OR	95% CI	*P* value	OR	95% CI	*P* value
Age	0.98	0.89–1.08	0.75	0.98	0.89–1.07	0.70
Gender	0.13	0.09–1.96	0.13	0.12	0.00-1.72	0.12
BMI	0.99	0.79–1.24	0.96	0.97	0.77–1.22	0.82
Current smoker	3.00	0.29–30.49	0.35	4.68	0.54-40.0	0.15
Antidepressant drugs	0.65	0.10–4.16	0.65	0.83	0.14–4.92	0.83
Social support	0.86	0.74–0.99	0.04*	0.88	0.77–1.01	0.07
PHQ-9 at baseline	1.09	0.96–1.25	0.17	1.09	0.95–1.25	0.18
TNF-α (pg/ml)	2.16	1.00-4.65	0.04*			
IL-1β (pg/ml)				1.26	0.90–1.78	0.17

OR, Odds ratio; 95% CI, 95% confidence interval; BMI, body mass index

*P < 0.05

### Patient characteristics

Patients were divided into four groups according to the interquartile interval of IL-1β or TNF-α. There were no significant differences in age, gender, BMI, education, etc. between four groups (*P* > 0.05). The only significant difference observed at the baseline assessment between the groups was that patients in married had lower levels of IL-1β (*P* = 0.01) (Table 3), and diabetes was associated with lower levels of TNF-α (*P* = 0.04) (Table 4).

**Table 3:** Baseline characteristics of patients stratified by IL-1β

Variable	IL-1β				*P* value
	< 25% (21)	25–50% (20)	50–75% (21)	> 75% (20)	
Age, year	39.1 ± 12.1	38.2 ± 12.5	34.6 ± 8.0	37.0 ± 15.1	0.66
Male gender	9 (42.8)	4 (20.0)	6 (28.5)	7 (35.0)	0.44
BMI, kg/m^2^	22.9 (8.5)	23.7 (4.4)	23.4 (5.8)	22.7 (5.0)	0.55
Married	17 (81.0) ^a^	12 (60.0) ^a,b^	13 (61.9) ^a,b^	6 (30.0) ^b^	0.01*
Education (≥ high school)	17 (81.0)	16 (80.0)	20 (95.2)	18 (90.0)	0.41
Employed	13 (61.9)	10 (50.0)	17 (81.0)	12 (60.0)	0.21
Income (RMB per year)					0.36
< 50,000 50,000-100,000 > 100,000 Unknown	5 (23.8) 7 (33.3) 8 (38.1) 1 (4.8)	9 (45.0) 2 (10.0) 8 (40.0) 1 (5.0)	2 (9.5) 5 (23.8) 11 (52.4) 3 (14.3)	4 (20.0) 5 (25.0) 9 (45.0) 2 (10.0)	
Current smoker	4 (19.0)	4 (20.0)	5 (23.8)	4 (20.0)	1.00
Current drinker	5 (23.8)	9 (45.0)	7 (33.3)	6 (30.0)	0.53
Physical activity					0.39
None Minor Moderate Severe	4 (19.0) 13 (61.9) 4 (19.0) 0 (0.0)	8 (40.0) 6 (30.0) 6 (30.0) 0 (0.0)	8 (38.1) 8 (38.1) 4 (19.0) 1 (4.8)	8 (40.0) 6 (30.0) 4 (20.0) 2 (10.0)	
Hypertension	5 (23.8)	7 (35.0)	1 (4.8)	2 (10.0)	0.05
Diabetes	3 (14.3)	0 (0.0)	0 (0.0)	0 (0.0)	0.05
Hyperlipidemia	4 (19.0)	4 (20.0)	3 (14.3)	5 (25.0)	0.85
Heart disease	3 (14.3)	2 (10.0)	2 (9.5)	2 (10.0)	1.00
Brain stroke	0 (0.0)	0 (0.0)	0 (0.0)	1 (5.0)	0.48
Tumor	0 (0.0)	0 (0.0)	1 (4.8)	1 (5.0)	0.86
Insomnia	12 (57.1)	11 (55.0)	14 (66.7)	7 (35.0)	0.22
Sleep apnea hypopnea syndrome	2 (9.5)	2 (10.0)	1 (4.8)	0 (0.0)	0.68
Family history of mental disease	2 (9.5)	2 (10.0)	4 (19.0)	6 (30.0)	0.29
Duration of depression	248 (729)	319.5 (837)	208 (1554)	82 (1418)	0.93
Antidepressant drugs	14 (66.7)	8 (40.0)	11 (52.4)	13 (65.0)	0.28
Social support	33.2 ± 8.4	30.2 ± 7.2	31.6 ± 9.8	31.3 ± 8.3	0.71
Life stress	160.6 ± 124.0	247.9 ± 133.5	234.8 ± 115.9	217.7 ± 123.2	0.16
PHQ-9	9.0 (11.0)	14.5 (10.0)	16.0 (9.0)	12.0 (3.0)	0.05
IL-1β	0.53 (0.73)	1.33 (0.36)	2.24 (0.74)	3.63 (2.56)	< 0.01*

IL-1β is stratified by quartile

All variables are expressed as number (percentage) except age, BMI, duration of depression, social support, life stress, PHQ-9 and IL-1β

^a^ and ^b^ mean difference between groups

BMI, body mass index

*P < 0.05

**Table 4:** Baseline characteristics of patients stratified by TNF-α

Variable	TNF-α				*P* value
	< 25% (21)	25–50% (21)	50–75% (20)	> 75% (20)	
Age, year	36.6 ± 11.2	37.2 ± 13.1	38.5 ± 12.1	36.6 ± 12.5	0.95
Male gender	8 (38.1)	7 (33.3)	6 (30.0)	5 (25.0)	0.83
BMI, kg/m^2^	23.7 (5.2)	24.1 (3.9)	22.8 (4.5)	21.5 (6.6)	0.47
Married	12 (57.1)	13 (61.9)	14 (70.0)	9 (45.0)	0.43
Education (≥ high school)	18 (85.7)	18 (85.7)	18 (90.0)	17 (85.0)	1.00
Employed	14 (66.7)	12 (57.1)	10 (50.0)	16 (80.0)	0.22
Income (RMB per year)					0.85
< 50,000 50,000-100,000 > 100,000 Unknown	5 (23.8) 4 (19.0) 10 (47.6) 2 (9.5)	6 (28.6) 6 (28.6) 9 (42.9) 0 (0.0)	5 (25.0) 3 (15.0) 9 (45.0) 3 (15.0)	4 (20.0) 6 (30.0) 8 (40.0) 2 (10.0)	
Current smoker	3 (14.3)	5 (23.8)	3 (15.0)	6 (30.0)	0.59
Current drinker	8 (38.1)	6 (28.6)	5 (25.0)	8 (40.0)	0.69
Physical activity					0.63
None Minor Moderate severe	6 (28.6) 10 (47.6) 3 (14.3) 2 (9.5)	7 (33.3) 6 (28.6) 7 (33.3) 1 (4.8)	8 (40.0) 7 (35.0) 5 (25.0) 0 (0.0)	7 (35.0) 10 (10.0) 3 (15.0) 0 (0.0)	
Hypertension	3 (14.3)	3 (14.3)	7 (35.0)	2 (10.0)	0.22
Diabetes	5 (23.8) ^a,b^	3 (23.8) ^a,b^	6 (30.0) ^b^	0 (0.0) ^a^	0.04*
Hyperlipidemia	1 (4.8)	2 (9.5)	0 (0.0)	0 (0.0)	0.61
Heart disease	2 (9.5)	3 (14.3)	2 (10.0)	2 (10.0)	1.00
Brain stroke	0 (0.0)	0 (0.0)	1 (5.0)	0 (0.0)	0.48
Tumor	0 (0.0)	0 (0.0)	1 (5.0)	1 (5.0)	0.36
Insomnia	13 (61.9)	12 (57.1)	10 (50.0)	9 (45.0)	0.70
Sleep apnea hypopnea syndrome	3 (14.3)	1 (4.8)	1 (5.0)	0 (0.0)	0.38
Family history of mental disease	1 (4.8)	5 (23.8)	3 (15.0)	5 (25.0)	0.25
Duration of depression	363 (828)	437 (1161)	297 (1842)	33.5 (733)	0.11
Antidepressant drugs	11 (52.4)	14 (66.7)	8 (40.0)	13 (65.0)	0.28
Social support	32 (15)	30 (13)	33 (10)	28.5 (16)	0.56
Life stress	214.3 ± 125.9	220.4 ± 133.5	215.4 ± 145.2	214.1 ± 101.5	0.99
PHQ-9	12.0 (10.0)	12.0 (13.0)	16.0 (10.0)	12.0 (4.0)	0.61
TNF-α	1.1 (0.2)	1.7 (0.4)	2.3 (0.4)	3.7 (1.6)	< 0.01*

TNF-α is stratified by quartile

All variables are expressed as number (percentage) except age, BMI, duration of depression, social support, life stress, PHQ-9 and TNF-α

^a^ and ^b^ mean difference between groups

BMI, body mass index

*P < 0.05

## Discussion

We reported that the IL-1β level at baseline was positively correlated with the severity of depression at middle-term follow-up. Higher TNF-α level and poor social support predicted a high risk of suicidal ideation and behavior. Married status was associated with lower levels of IL-1β, and diabetes was associated with lower levels of TNF-α.

Plasma IL-1β levels are significantly associated with depression and depressive severity [29], and also related to treatment-refractory depression [5]. The treatment response of depression is associated with specific genetic variants and methylation status of the gene IL-1β [30]. Higher level of IL-1β mRNA molecules predicts poor antidepressant response [31]. And IL-1β decreases after one month antidepressant treatment [32]. IL-1β, produced by macrophages, endothelial cells and astrocytes, can cross the blood brain barrier and alter the HPA-axis [33]. Within brain tissue, microglia expresses the P2X7 ion channel, and releases IL-1β through NLRP3 inflammasome complex. The P2X7-NLRP3-IL-1β pathway is associated with depression [34]. IL-1β also elevated in hippocampus. TLR4-NF-κB/NLRP3/ IL-1β pathway may be a key signaling pathway in depression [35]. IL-1β can suppress vitro neurogenesis of human hippocampal progenitor cells [36], induce synaptic pruning, which leads to impaired neuroplasticity and structural brain changes [5], both are common in depression. It can induce the expression of the reuptake transporter for serotonin by p38 mitogen-activated protein kinase, resulting in a poor efficacy of traditional antidepressants [37]. Minocycline can ameliorate depression by reducing the levels of IL-1β in the hippocampus [38]. Here we found IL‑1β had predictive effect on middle-term prognosis of depression, which maybe an interventional target or predictive factor.

Plasma TNF-α, a pro-inflammatory cytokine produced by macrophages, adipocytes and astrocytes, is highly positively correlated with depressive subjects and greater depressive symptom [5, 39]. Depression with high levels of TNF-α stimulates NF-κB, then elevates histone deacetylase 1 activity and represses claudin-5 expression, resulting in loss of tight-junction proteins and disruption of blood-brain barrier [40]. It also transports BBB via TNF-α receptors without BBB disruption [41]. High levels of TNF-α in dorsolateral prefrontal cortex, striatum and hippocampus are also associated with depressed behavior [42–44]. Only a little data shows slightly reduced TNF-α level in depression [45]. TNF-α level may rise at the onset of depression for compensating neuronal disturbances and decrease after antidepressant treatment [45]. High levels of plasma or prefrontal cortex TNF-α is associated with suicidal ideation in depression [46, 47] or non-depressed people [44]. While there is also report shows that suicidal adolescents with depression have lower plasma TNF-α levels than non-suicidal one [5]. And some reports show TNF-α is not associated with suicide [48]. In this report, we found high TNF-α level predicted suicidal ideation and behavior. It is possible that the underlying pathophysiology differs between adolescents and adults. The cytokine levels increase with age. In the late-life depression, TNF-α contributes to the reduction of serotonin [11]. TNF-α triggers degradation of NIP3-like protein X, an outer mitochondrial membrane protein, and then impairs the mitophagy in the medial prefrontal cortex [49]. TNF-α alters serotonin metabolism by inducing the tryptophan-metabolising enzyme indoleamine 2, 3-dioxygenase [50, 51], and augmenting the activity of serotonin transporters. It also overactivates the HPA axis [52], and is correlated with 5-hydroxyindolacetic acid and homovanillic acid [44]. All are thought to be persistent symptoms in suicide.

Depressive patients lack of social support predict greater depression severity [53], and have worse prognoses than those with good social support [54]. Social isolation and social support seem to appear respectively as risk and protective factors for suicide [55]. Spouses are the primary social supports, married individuals are less depressed than unmarried [56]. Negative social interactions may increase the levels of pro-inflammatory cytokines, such as IL-6, TNF-α [24], and positive social support can activate opioid system and produce beta-endorphin that has anti-inflammatory properties, which are associated with depression severity and suicide [25]. We found better social support predicted low suicidal ideation and behavior risk, and married patients had lower levels of IL-1β. Inflammatory response may be a potential mechanism for poor social support mediates depression. We found that diabetes was associated with lower levels of TNF-α. Antidiabetic drugs may play an anti-inflammatory role in diabetics [57]. Unfortunately, we did not collect information of diabetes drugs.

Our study presents several limitations. First, the sample size was small and all samples were from a single center. Second, some patients had taken medicine at the baseline, while we adjusted this factor in model 2. Third, we didn’t collect the data on number of previous lifetime depressive episodes and suicidal behavior, and number of previous antidepressant used in the current episode. Fourth, our study couldn’t determine how these makers change in healthy controls, and didn’t measure inflammatory cytokines during follow-up. Fifth, eleven patients were lost during follow-up. We found this group of patients had a low level of education and high proportion of heart disease, and they also participated less in physical activity than those who completed follow-up. There were no significant differences in age, gender, BMI, etc.

## Conclusions

In this study, we found a predictive role of IL-1β for symptomatic improvement after 3-month follow-up of depressive patients. Compared with the patients with high levels of IL-1β, the low one had milder depressive severity at 2-month and 3-month follow-up. In addition, baseline TNF-α might predict middle-term suicidal ideation and behavior in depression patients.

### Electronic supplementary material

Below is the link to the electronic supplementary material.

Supplementary Material 1

Supplementary Material 2

## Data Availability

The raw data supporting the conclusions of this article will be made available by Fei Liu (liufeifay@163.com), without undue reservation.

## Abbreviations

IL: Interleukin
IFN: Interferon
TNF: Tumor necrosis factor
TGF: Tumor transforming growth factor
HPA: Hypothalamic-pituitary-adrenal
BBB: Blood brain barrier
CNS: Central nervous system
CRH: Corticotropin releasing hormone
ACTH: Adrenocorticotropic hormone
5-HT: 5-hydroxytryptamine
SSRI: Selective serotonin and noradrenaline reuptake inhibitors
SNRI: Serotonin and noradrenaline reuptake inhibitors
TCA: Tricyclic antidepressants
PHQ-9: Patient Health Questionnaire-9
BMI: Body mass index
IQR: Interquartile range
VIF: Variance inflation factor
OR: Odds ratios
