# Supplementary material for: Impacts of inflammatory cytokines on depression: a cohort study
Source: BMC Psychiatry. 2024 Mar 8;24:195. doi: 10.1186/s12888-024-05639-w (PMC10924400; doi:10.1186/s12888-024-05639-w)
Supplement: Supplementary file 1 — Supplementary Material 1 [file 12888_2024_5639_MOESM1_ESM.docx]

**Supplemental table 1.** Linear regression analysis for the baseline IL-1β and depression at month 2

|  | 2-month PHQ-9 | | |
| --- | --- | --- | --- |
|  | B | P value | VIF |
| Age | -0.05 | 0.40 | 1.53 |
| BMI | -0.04 | 0.79 | 1.45 |
| Social support | -0.12 | 0.17 | 1.49 |
| Life stress | 0.00 | 0.65 | 1.49 |
| PHQ-9 at baseline | 0.34 | **<0.01** | 1.34 |
| Education | -0.15 | 0.94 | 1.22 |
| Antidepressant drugs | 0.42 | 0.76 | 1.05 |
| IL-1β (pg/ml) | 0.92 | **<0.01** | 1.16 |

PHQ-9, Patient Health Questionnaire-9; VIF, variance inflation factor; BMI, body mass index
