# Supplementary material for: Impacts of inflammatory cytokines on depression: a cohort study
Source: BMC Psychiatry. 2024 Mar 8;24:195. doi: 10.1186/s12888-024-05639-w (PMC10924400; doi:10.1186/s12888-024-05639-w)
Supplement: Supplementary file 2 — Supplementary Material 2 [file 12888_2024_5639_MOESM2_ESM.docx]

**Supplemental table 2.** Linear regression analysis for the baseline IL-1β and depression at month 3

|  | 3-month PHQ-9 | | |
| --- | --- | --- | --- |
|  | B | P value | VIF |
| Age | -0.06 | 0.38 | 1.50 |
| BMI | -0.06 | 0.72 | 1.42 |
| Social support | -0.09 | 0.36 | 1.48 |
| Life stress | 0.00 | 0.96 | 1.46 |
| PHQ-9 at baseline | 0.34 | **<0.01** | 1.34 |
| Education | -0.12 | 0.96 | 1.23 |
| Antidepressant drugs | -1.85 | 0.22 | 1.02 |
| IL-1β (pg/ml) | 0.86 | **0.02** | 1.17 |

PHQ-9, Patient Health Questionnaire-9; VIF, variance inflation factor; BMI, body mass index
